# Energy Balance Regulating Neuropeptides Are Expressed through Pregnancy and Regulated by Interleukin-6 Deficiency in Mouse Placenta

**DOI:** 10.1155/2014/537603

**Published:** 2014-03-12

**Authors:** Patricia Pazos, Luis Lima, Carlos Diéguez, María C. García

**Affiliations:** ^1^Department of Physiology, Research Center of Molecular Medicine and Chronic Diseases (CIMUS), University of Santiago de Compostela, Avenida de Barcelona s/n, 15782 Santiago de Compostela, Spain; ^2^Instituto de Investigación Sanitaria de Santiago de Compostela (IDIS), 15706 Santiago de Compostela, Spain; ^3^CIBER Fisiopatología Obesidad y Nutrición (CB06/03), Instituto de Salud Carlos III (ISCIII), Ministerio de Economía y Competitividad (MINECO), 15706 Santiago de Compostela, Spain

## Abstract

The placenta produces a number of signaling molecules including metabolic and reproductive hormones as well as several inflammatory mediators. Among them, Interleukin-6 (IL-6), a well-known immune and metabolic regulator, acts peripherally modulating metabolic function and centrally increasing energy expenditure and reducing body fat. IL-6 interacts with key hypothalamic neuropeptidergic systems controlling energy homeostasis such as those producing the orexigenic/anabolic: neuropeptide Y (NPY) and agouti-related peptide (AgRP) and anorectic/catabolic neuropeptides: proopiomelanocortin (POMC) and cocaine and amphetamine regulated transcript (CART). Human and rat placenta have been identified as source of these neuropeptides, but their expression and regulation in murine placental tissues remain unknown. Therefore, placental mRNA levels of IL-6, NPY, AgRP, POMC, and CART at different pregnancy stages (gestational days 13, 15, and 18) were analyzed by real time PCR, as were the effect of IL-6 deficiency (IL-6 knockout mice) on their placental expression. Our results showed that placenta-derived neuropeptides were regulated by gestational age and IL-6 throughout the second half of mouse pregnancy. These data suggest that IL-6 may participate in the fine tune control of energy balance during pregnancy by extending its action as a metabolic signal to the main organ at the fetomaternal interface: the placenta.

## 1. Introduction

Energy homeostasis, defined as the process whereby body weight and energy reserves are maintained stable over long periods of time, is tightly regulated through the complex interactions of the brain and peripheral organs [1, 2]. Reciprocal neural networks within the hypothalamus and the brain stem act in response to peripheral (metabolic, endocrine, and neural) signals of metabolic status, matching energy intake with energy outputs such as basal metabolism, activity, thermogenesis, and reproduction [3–5].

Afferent endocrine signals projecting to the brain to encode short- and long-term energy status comprise a wide variety of tissue-specific signalling molecules including: (i) adipokines (e.g., leptin, adiponectin, resistin, and interleukin-6 (IL-6)), secreted by the expanding adipose tissue; (ii) pancreatic hormones such as insulin and amylin; (iii) gastrointestinal peptides, such as ghrelin, glucagon-like peptide I, and peptide YY; and (iv) gonadal and placental hormones, such as steroids and placental lactogens [2, 6–8]. Several hypothalamic neuronal populations express specific receptors for these metabolic hormones and communicate within each other via chemical synapses and the release of neurotransmitters and neuropeptides, characterized by having either direct or indirect effects on body weight regulation and reproductive function [9].

Two opposing cell types in the arcuate nucleus (ARC) are a relevant example of the integrative nature of the hypothalamus in the regulation of energy metabolism and fertility [10]. One type produces the orexigenic and anabolic neuropeptide Y (NPY) and agouti-related peptide (AgRP) as well as gamma-aminobutyric acid (GABA). The other produces cocaine and amphetamine regulated transcript (CART) and proopiomelanocortin- (POMC-) derived peptides, such as alpha melanocyte stimulating hormone (*α*MSH), that promote anorexia by inhibiting food intake and increases catabolic processes. *α*MSH modulates its downstream homeostatic signalling via their action at melanocortin receptors MC3R and MC4R, which are antagonized by AgRP [2]. The coordinated regulation of these neurons and their efferent projections to key brain regions such as the paraventricular nucleus (PVN) and the medial preoptic and lateral hypothalamic area (LH) contributes to regulate energy metabolism, gonadotropin-releasing hormone (GnRH) release, and pituitary-gonadal axis activity [2, 10, 11].

The placenta, a unique and autonomous transient organ, represents the primary immunological and nutrient transport barrier between the mother and the fetus. Throughout its entire lifespan, the placenta is also able to produce as well as respond to a variety of signalling molecules required for pregnancy establishment and maintenance but also for maternal adaptation to pregnancy, as well as fetal growth and development. Placental secretory factors include among others: steroid hormones, growth factors, metabolic hormones (e.g., leptin [12, 13], adiponectin [14, 15] and resistin [16, 17]), and pro- and anti-inflammatory cytokines as interleukins-6 and -10 (IL-6 and IL-10) [18, 19]. Despite the lack of innervation in this organ, production of energy balance regulating neuropeptides has been reported in rat [20, 21] and human [22–25] placenta, where they may play a regulatory role in placental hormonal secretion that affects maternal and fetal metabolism. However, the physiological relevance and regulation of these placental neuropeptides remain unclear.

IL-6 is a pluripotent cytokine not only involved in the immune response, but with multiple relevant effects on many different cell types including those of the nervous [26] and reproductive systems [27, 28]. Recent evidence from our group [29] and others [30–32] points to the IL-6 system as a key pathway involved in the central regulation of food intake and energy metabolism. Hence, centrally (astrocyte-restricted) [26] and total IL-6 deficiency in mice [33–35] are associated with increased body weight and adiposity at young and late ages, while long-term central (intracerebroventricular) but not peripheral IL-6 treatment exerts a restorative effect on fat mass values [36]. The antiobesity IL-6 action likely involves enhanced thermogenesis and lipid oxidation rather than reduced food intake [29, 37–39]. To control this physiological functions in health [29, 38, 40] and disease conditions [41], IL-6 engages its widely expressed specific receptor IL-6 receptor alpha [42] to modulate the expression of key anabolic and catabolic hypothalamic neuropeptides at the level of the ARC, PVN, and LH [31, 42, 43].

Our group has recently demonstrated that pregnancy in the mouse is associated with a progressive increase in circulating IL-6 levels, while hypothalamic IL-6 and IL-6Ra expression are depressed [29]. These changes in the central and peripheral expression of the IL-6 system are accompanied by specific adaptations in the dam's hypothalamic circuits governing energy balance, notably at the level of the ARC and PVN. Major sources of circulating IL-6 during this physiological state include the expanding adipose tissue as well as the placenta, where it has been related to disarrangements in maternal-fetal nutrient (i.e., lipid) transfer in complicated human pregnancies [44]. However, little is known about the possible influence of this cytokine on the placental production of energy balance regulating neuropeptides. Thus, the present study was designed to determine whether during the second half of pregnancy NPY, AgRP, POMC, and CART mRNA are expressed and ontogenetically modulated in the mouse placenta. Using interleukin-6 knockout mice (IL-6 KO mice) as a model, the effects of IL-6 deficiency on their gestational pattern of placental expression were also investigated.

## 2. Materials and Methods

### 2.1. Animals

IL-6 deficient mice (B6.129S2-IL-6 tm1 kopf/J, sourced from Jackson Laboratories) [45] that had undergone eleven backcrosses to the C57BL/6 background and their wild-type congenic C57BL/6J controls were purchased from Jackson laboratories (Charles River, Barcelona, Spain; stock numbers 002650 and 000664, resp.). After weaning, animals were housed 5-6 per cage under controlled temperature conditions (22°C) and a 12-hour light/dark cycle with free access to water and rodent chow (2019s, Teklad Global, Harlan, Spain). Age-matched female WT and IL-6 KO mice (12 weeks old) were always used for experiments. Ethical approval was obtained from the University of Santiago de Compostela Bio-ethics Committee and all the procedures were conducted according to the regulations of the European Community for the care and use of experimental animals.

### 2.2. Experimental Design

To obtain pregnant animals, twelve-week-old female IL-6 KO or WT mice (*n* = 27 and 38, resp.) were mated for three days with stud mice of the same genotype. Successful matings, as judged by the presence of a vaginal plug in the early morning (day of occurrence = day 0 after coitum), were detected in a total of 22 IL-6 KO mice (81%) and 28 WT (74%). On the corresponding day of pregnancy (day 13, 15, or 18), timed pregnant animals were anesthetized and after collection of serum samples by cardiac puncture were sacrificed by decapitation. About 82 and 61% of the plugged IL-6 KO and WT mice were pregnant (*n* = 18 and 17, resp.), and only one dam of each genotype was excluded for the analysis due to presence of less than 7 viable (normal appearing) fetuses (Table 1). Placental samples were removed, weighed, frozen on dry ice, and stored in –80°C until further analysis. At each gestational age analyzed, the number of fetuses per litter was similar in WT and IL-6 KO groups as were the weights of conceptuses and placentas (Table 1). We used 5-6 placentas per experimental group, extracted from different mothers. All the samples were analyzed individually and samples were not pooled.

### 2.3. Serum Assays

Circulating serum levels of interleukin 6 were assayed by ELISA, using a commercial kit (IL-6, Abcam, Cambridge, UK) according to the manufacturer's instructions.

### 2.4. Real Time qPCR

Real-time quantitative RT-PCR (qPCR) analysis was carried out as previously described [46]. Total RNA was obtained by homogenization in TRIZOL reagent according to the manufacturer's instructions (Invitrogen, Barcelona, Spain). First-strand cDNA was synthesized from 1.5 *μ*g of total RNA in a 30 *μ*L reaction using 200 U Maloney murine leukemia virus reverse transcriptase and random hexamer primers (Invitrogen, Barcelona, Spain). qPCR was performed using a Mastercycler EP Realplex real-time PCR system (Eppendorf). Probe detection (Universal Master mix; Applied Biosystems) was used for IL-6 [47] and the neuropeptides NPY, AgRP, and CART expression analysis [48] (Table 2). SYBR Green was used for POMC mRNA detection [21] (Luminaris Color HiGreen qPCR Master Mix; Thermo Fisher Scientific) and was followed by a melting curve to assure the primers specificity (Table 2).

A standard curve was run in each assay, with an arbitrary value assigned to the highest standard and corresponding values to the subsequent dilutions. A nontemplate reaction was included during each experiment to control for DNA contamination. Mouse hypoxanthine guanine phosphoribosyl transferase (HPRT) was used as control house-keeping gene [49–51]. The relative expression levels were estimated using the comparative threshold cycle method [52] and all samples were normalized against HPRTexpression values. We have previously used HPRT as reference gene in the rat [14, 21] but not in the mouse placenta. Therefore, we tested whether the HPRT mRNA levels were stable in placentas from mice of the same genotype at different gestational ages. HPRT mRNA expression was similar among gestational age groups, hence validating its use as reference gene in the present study.

To verify the identity of amplified cDNAs, PCR products were electrophoresed on a 1.5% agarose gel, which yielded DNA fragments of the expected length for all mRNAs and were confirmed by sequencing (data not shown).

### 2.5. Statistical Analysis

Results are given as mean ± SEM. All data were analyzed using Graph Pad Prism version 6.00 for Windows (GraphPad Software, La Jolla, California USA). Comparisons between two groups were performed with unpaired two-tailed Student's *t*-test and one-way analysis of variance (ANOVA), followed by the Bonferroni post hoc test, when differences between more than two experimental groups were analyzed.

## 3. Results 

### 3.1. Increased Serum IL-6 Levels during Pregnancy in WT Mice

**Figure 1:**
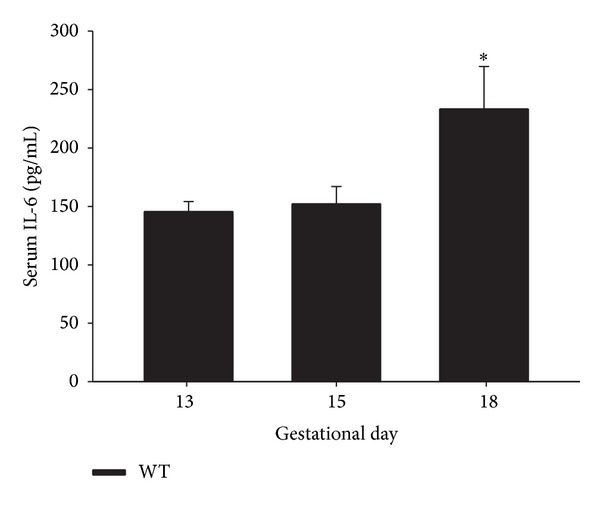
IL-6 levels in serum of WT (C57BL6) mice. 12-week-old WT mice were time-pregnant and serum IL-6 levels at different pregnancy stages (13, 15, and 18 days) were assessed by ELISA; *n* = 5-6. Each value represents the mean ± SEM. One-way ANOVA: *P < 0.05 versus 13 days pregnant.

Firstly, circulating levels of IL-6 were assessed during pregnancy. As expected [29, 53] serum IL-6 levels increased as pregnancy progressed although a significant stimulatory effect was only evident at gestational day 18 from values at gestational day 13 (*P* < 0.05) (Figure 1).

### 3.2. IL-6 mRNA Expression Is Up-Regulated in the Placenta of Pregnant WT Mice Throughout Pregnancy

**Figure 2:**
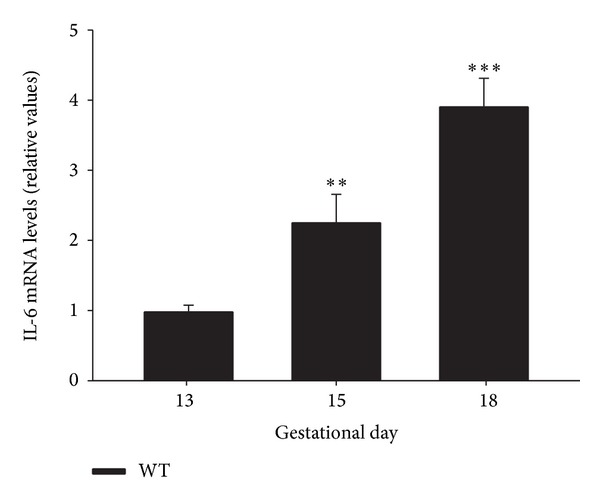
IL-6 mRNA expression in placenta of WT (C57BL6) mice. 12-week-old WT mice were time-pregnant and placental IL-6 mRNA levels at different pregnancy stages (13, 15, and 18 days) were assessed by real-time qPCR; *n* = 5-6. Relative mRNA levels were normalized to 13 days pregnant mice values as 1. Each value represents the mean ± SEM. One-way ANOVA: **P < 0.01 versus 13 days pregnant mice values.

Thereafter we analyzed the transcriptional profile of placental IL-6 expression during mouse pregnancy. IL-6 mRNA was detected in all placentas tested from pregnant WT dams (C57BL6 female mice) at gestational days 13, 15, and 18. Our data showed that IL-6 gene expression increased with gestational age in the mouse placenta, with significantly higher mRNA levels on days 15 and 18 of pregnancy in comparison to values on day 13 (*P* < 0.01 and *P* < 0.001, resp.) (Figure 2).

### 3.3. NPY mRNA Expression Is Up-Regulated by IL-6 Deficiency in the Mouse Placenta

**Figure 3:**
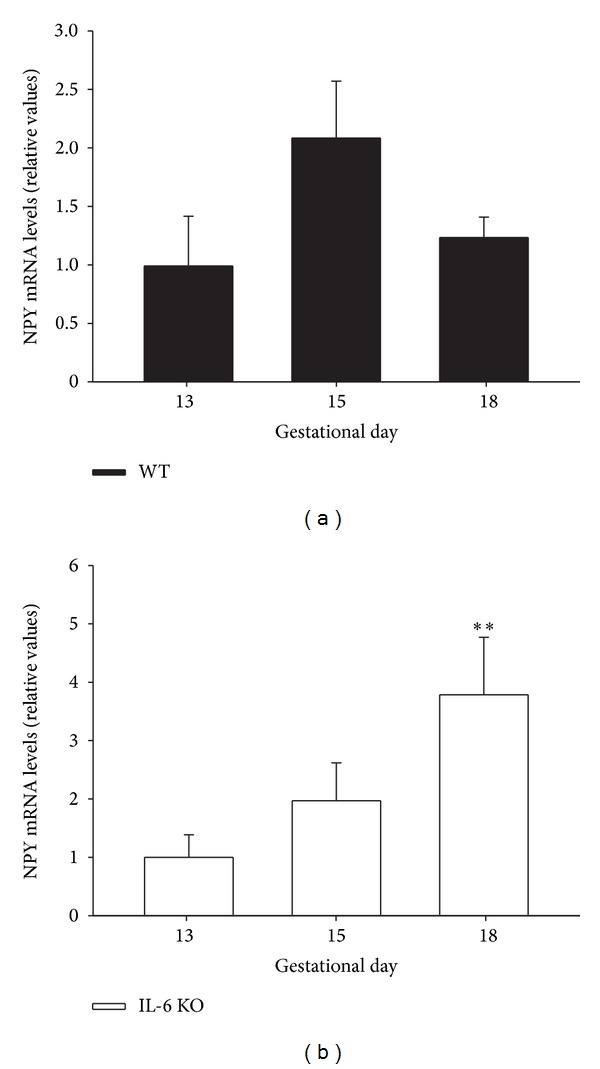
NPY mRNA expression in placenta of WT and IL-6 KO mice. 12-week-old WT and IL-6 KO mice were time-pregnant and placental NPY mRNA levels in (a) WT and (b) IL-6 KO mice at different pregnancy stages (13, 15, and 18 days) were assessed by real-time qPCR; *n* = 5-6. Within each genotype, relative mRNA levels were normalized to 13 days pregnant mice values as 1. Each value represents the mean ± SEM. One-way ANOVA: **P < 0.01 versus 13 days pregnant mice of respective genotype.

Next we evaluated the placental NPY mRNA levels and whether absence of IL-6 might influence its gestational profile during mouse pregnancy. Although, in the placenta of WT mice, there was a trend towards an increased NPY mRNA expression at gestational day 15 in relation to the other stages of pregnancy studied, no statistical difference was observed (Figure 3(a)). On the contrary, in absence of IL-6, placental NPY mRNA expression remained unchanged between 13 and 15 days of pregnancy reaching the highest values at the end of the gestational period (*P* < 0.01) (Figure 3(b)).

### 3.4. Lack of IL-6 Does Not Affect AgRP mRNA Expression in the Mouse Placenta

**Figure 4:**
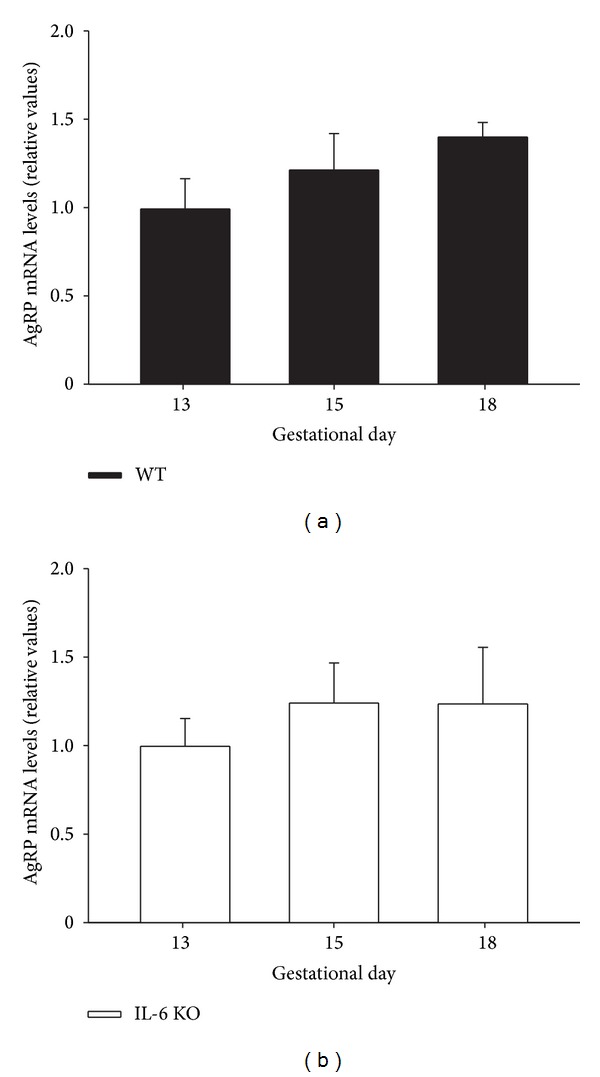
AgRP mRNA expression in placenta of WT and IL-6 KO mice. 12-week-old WT and IL-6 KO mice were time-pregnant and placental AgRP mRNA levels in (a) WT and (b) IL-6 KO mice at different pregnancy stages (13, 15, and 18 days) were assessed by real-time qPCR; *n* = 5-6. Within each genotype, relative mRNA levels were normalized to 13 days pregnant mice values as 1. Each value represents the mean ± SEM.

AgRP mRNA was expressed in the mouse placenta at all gestational ages assessed but showed a slightly different profile during pregnancy from that of NPY. Thus, AgRP mRNA content in the placenta of pregnant WT mice did not change significantly from 13 to 18 days of pregnancy (Figure 4(a)), being unaffected by IL-6 deficiency (Figure 4(b)).

### 3.5. POMC mRNA Expression Is Down-Regulated by IL-6 Deficiency in the Mouse Placenta

**Figure 5:**
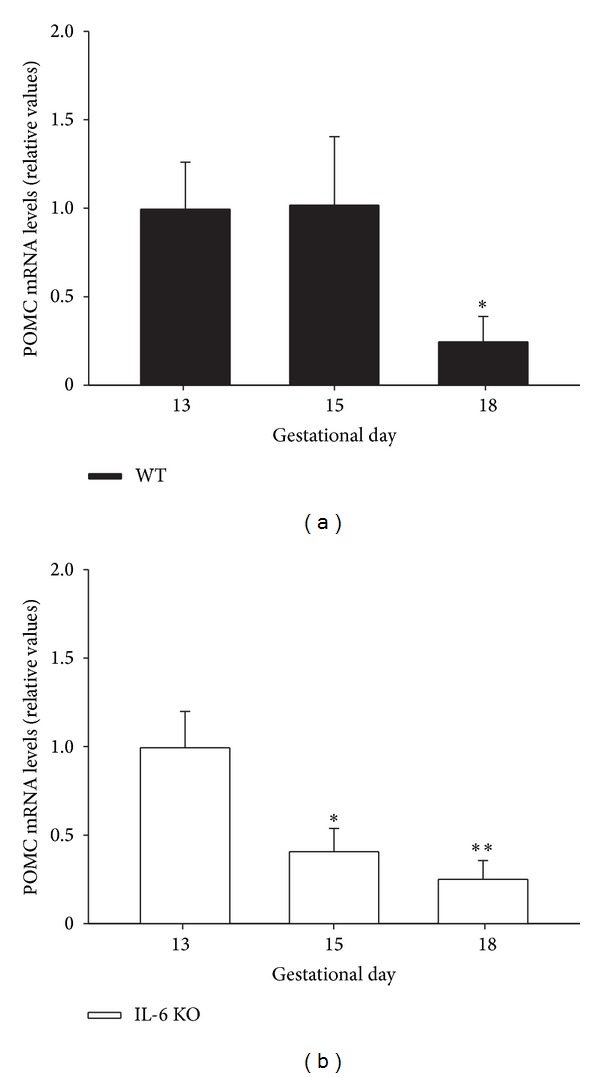
POMC mRNA expression in placenta of WT and IL-6 KO mice. 12-week-old WT and IL-6 KO mice were time-pregnant and placental POMC mRNA levels in (a) WT and (b) IL-6 KO mice at different pregnancy stages (13, 15, and 18 days) were assessed by real-time qPCR; *n* = 5-6. Within each genotype, relative mRNA levels were normalized to 13 days pregnant mice values as 1. Each value represents the mean ± SEM. One-way ANOVA: *P < 0.05, **P < 0.01 versus 13 days pregnant mice of respective genotype.

Placental POMC mRNA expression in WT mice was markedly reduced at the latest pregnancy stage (gestational day 18, *P* < 0.05), while it remained unchanged from 13 to 15 days of pregnancy (Figure 5(a)). However, IL-6 KO mice showed a significantly (*P* < 0.05) reducing trend for placental POMC mRNA content from gestational days 13 to 18 (Figure 5(b)).

### 3.6. CART mRNA Expression Is Up-Regulated by IL-6 Deficiency in the Mouse Placenta

**Figure 6:**
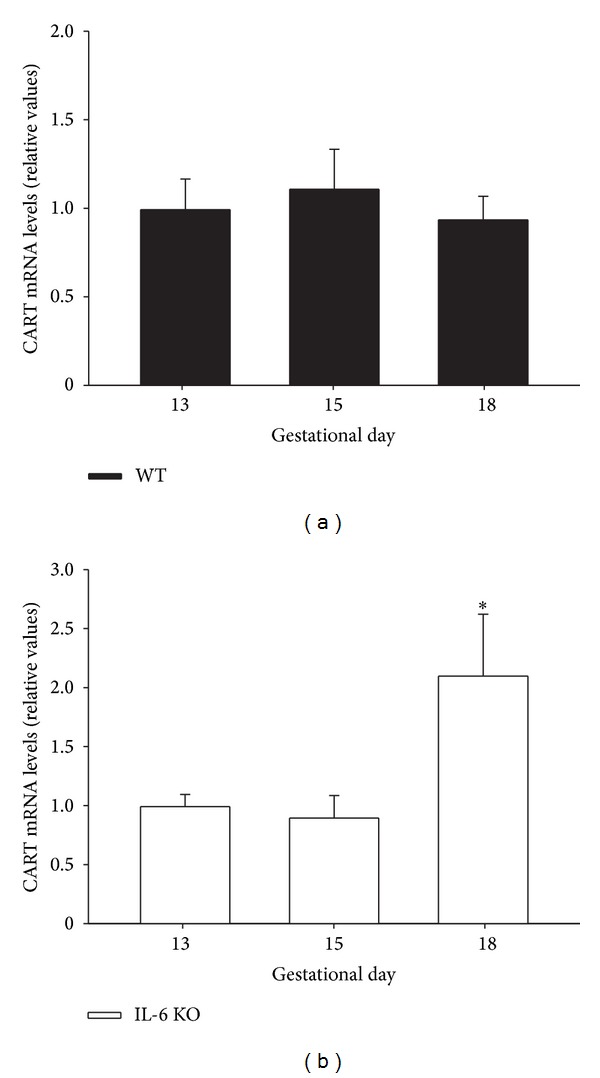
CART mRNA expression in placenta of WT and IL-6 KO mice. 12-week-old WT and IL-6 KO mice were time-pregnant and placental CART mRNA levels in (a) WT and (b) IL-6 KO mice at different pregnancy stages (13,15, and 18 days) were assessed by real-time qPCR; *n* = 5-6. Within each genotype, relative mRNA levels were normalized to 13 days pregnant mice values as 1. Each value represents the mean ± SEM. One-way ANOVA: *P < 0.05 versus 13 days pregnant mice of respective genotype.

Finally, we studied the transcriptional profile of CART expression in the placenta of WT and IL-6 KO mice during pregnancy. Our data showed that this catabolic neuropeptide had a constitutive and fairly constant expression in the placenta of WT mice throughout pregnancy (Figure 6(a)). A similar pattern was observed in the placentas from IL-6 KO mice until gestational day 18, when the highest levels of placental CART mRNA expression were reached (*P* < 0.01) (Figure 6(b)).

## 4. Discussion

Pregnancy is now recognized as a state of adaptive, low-grade-inflammation induced by a progressive increase of circulating levels inflammatory mediators such as cytokines, chemokines, and acute-phase reactants [53, 54]. Cytokines, central players of this inflammatory response, are secreted by the immune cells and adipocytes but also by placental cells [55]. Placental cytokine production and action are crucial to control trophoblast implantation, embryo development, and feto-maternal tolerance [55]. However, once pregnancy is established, the physiological relevance of most placental cytokines remains unclear.

Proinflammatory cytokines, such as IL-6, TNF alpha, and IL-1, are known to negatively influence peripheral lipid and glucose metabolism. Hence, by secreting these immune factors, the placenta may enhance the contribution made by adipose tissue to induce maternal insulin resistance and ensure a proper glucose transfer to the growing fetus. In this study, we found increased expression of IL-6 mRNA in the mouse placenta during gestation. These results are in agreement with previous reports of an enhanced IL-6 placental production during the latest stages of rat [54] and human pregnancies [56]. To our knowledge, the present work is the first one to determine the ontogenetic profile of IL-6 mRNA levels in the mouse placenta. In fact, De and coworkers [57] showed high levels of placental IL-6 bioactivity but failed to demonstrate its constitutive transcriptional expression in this mouse reproductive organ. The reason for this discrepancy may be the different methodologies employed, since in this previous study IL-6 mRNA levels were measured by Northern blot while we used a more sensitive technique, RT-qPCR. We [29] and others [53, 58] have previously found elevated IL-6 serum and adipose tissue mRNA levels during mouse late pregnancy. Our data do not allow us to determine the relative contribution of both tissues to the amount of this cytokine present in maternal circulation, but the similar pattern of IL-6 mRNA overexpression in both tissues suggests that both could be a source of circulating IL-6 in pregnancy.

Our group has recently shown that, in addition to its peripheral metabolic effects, IL-6 modulates the central mechanisms associated to maternal hyperphagia and increased adiposity in the pregnant mouse [29]. Although the transcriptional expression of its hypothalamic neuropeptide targets NPY, AgRP, POMC, and CART in the human [24, 25] and rat placenta [20] is well established, whether they are also placentally produced and regulated by IL-6 during mouse pregnancy remains unknown. In order to clarify this issue, we determined the placental mRNA expression of these energy balance regulating neuropeptides throughout pregnancy (from gestational days 13 to 18) in WT and IL-6 deficient mice.

The results of the present study revealed a similar transcriptional regulation of ARC neuropeptides in the mouse placenta from that previously reported in the hypothalamus [29]. Thus, POMC mRNA levels were reduced from gestational days 13 to 18, while the expression of the orexigenic and anabolic neuropeptides NPY/AgRP remains unchanged. A similar absence of relation between gestational age and placental NPY mRNA content has been shown in other species [20, 25]. On the contrary, the placental ontogenetic expression of the melanocortin system members POMC and AgRP seems to be a rather controversial issue. Therefore, some studies reported a decrease in AgRP mRNA levels with gestational age and no change in POMC levels, whilst others showed an upregulated mRNA expression of both neuropeptides in human [24] and rat placentas [20, 21]. As far as we are aware, the mRNA expression of CART has been previously examined neither in human nor in murine placental tissues. In this sense, recent data from our group demonstrated that the mRNA levels of this catabolic and anorectic neuropeptide are markedly increased in the rat placenta at term [20, 21]. However, in the present study we failed to detect any change in CART gene expression throughout mouse pregnancy. The reason for this discrepancy is unclear but it might reflect some biological differences between species.

Another novel finding of the present study is the fact that IL-6, acting on an autocrine or paracrine manner, modulates the placental expression of classical ARC energy balance regulatory peptides, as shown by IL-6 KO mice data. Thus, NPY and CART mRNA levels are upregulated by IL-6 deficiency at late pregnancy while POMC gene expression is downregulated in placentas from 15-day pregnant IL-6 KO mice. These results indicate that, during the third week of pregnancy, lack of IL-6 settles an anabolic milieu in the mouse placenta by reciprocally modulating the transcriptional expression of neuropeptides known to either promote, NPY, or repress, POMC, net energy gain. As we have previously demonstrated in the hypothalamus of the mouse in late pregnancy a lack of IL-6 upregulates NPY/AgRP mRNA levels in the ARC; it seems that IL-6 deficiency during late pregnancy exerts a stimulatory effect on NPY gene transcription whatever is its source. However, the results obtained regarding placental CART/POMC gene expression were somewhat unexpected. Increased hypothalamic CART or POMC production has been reported after endotoxemic challenge or central administration of other well-known proinflammatory cytokines as IL-1 [59, 60]. In addition, exercise-induced hypothalamic IL-6 and IL-10 in obese rats [61] and IL-6 astrocyte-targeted overexpression in female mice have been shown to increase POMC expression in the ARC [62]. Conversely, total knockout of IL-6 in mice restored to virgin values the reduced POMC mRNA levels in the ARC of late pregnant mice, probably reflecting a compensatory mechanism exerted by other factors [29]. On the contrary, the data reported herein indicate that, during the second half of pregnancy, this compensatory effect on POMC gene expression is not settled in the mouse placenta. Considering the above mentioned results and the fact that CART and POMC are colocalized in the ARC, a similar pattern of placental expression might have been expected for both neuropeptides. However, we have not performed in situ hybridization or immunohistochemical studies to characterize cellular distribution of CART and POMC in placental tissue. Therefore, we cannot exclude the possibility that different placental cell types could produce these neuropeptides and respond to absence of IL-6 in a different manner. In fact, with respect to their hypothalamic regulation, it is worth to mention that CART mRNA has been reported in a number of hypothalamic cell populations besides the ARC, where its expression has been shown to be differentially modulated from that of POMC in response to endotoxin challenge [63].

Finally, in relation to the previous point, it is important to take into account that the experimental approach used in the current study was designed to assess, during the second half of pregnancy, global changes in IL-6, and main energy regulating neuropeptides gene expression in the mouse placenta. Additionally, the IL-6 deficient mouse was chosen to address the influence of total absence of endogenous IL-6 on this setting. However, the murine placenta consists of three mayor cell layers of maternal or placental origin: the outer maternal decidua, the middle junctional zone, composed of fetoplacental trophoblast cells, and the inner labyrinth zone [64]. Therefore further in vitro studies involving either decidual or trophoblast cells in culture would be helpful to determine the ability of each cell population to produce and secrete these neuropeptides and confirm the contribution of IL-6 to their transcriptional modulation.

Whatever is the case; these results of the current study suggest that IL-6, by exerting a direct or indirect regulatory action in the transcriptional control of NPY, CART, and POMC expression, may play a role in the homeostatic response to energy availability in mouse placenta. Finally, whether placental neuropeptide protein levels correlate with the ontogenetic neuropeptide mRNA pattern herein shown will merit further investigation.

## 5. Conclusions 

In summary, we demonstrate that as in other species, the classical hypothalamic neuropeptidergic systems involved in energy homeostasis are present in the mouse placenta. These central signals display a specific ontogenetic expression pattern in absence of IL-6 with major changes settled in placentas at term. Thus, our results suggest that IL-6, a well-known immune and metabolic modulating factor, might modulate their transcriptional expression by acting on an autocrine or paracrine manner. Collectively, these results suggest that during an energetic challenging condition such as pregnancy IL-6 may also extend its action as a metabolic signal to the main organ at the maternal-fetal interface: the placenta.

## Figures and Tables

**Table 1:** Litter size, weights of dams, conceptuses, and placentas in WT and IL-6 KO dams.

Gestational day	Dam weight (g)		Conceptus weight (mg)		Placental weight (mg)		Litter size	
	WT	IL-6 KO	WT	IL-6 KO	WT	IL-6 KO	WT	IL-6 KO
13	29.87 ± 0.57	28.15 ± 0.49*	410 ± 64	421 ± 41	77 ± 13	72 ± 9	8.8 ± 0.8	8.5 ± 0.8
	(6)	(6)						
15	34.61 ± 0.72	31.67 ± 0.68*	756 ± 54	696 ± 37	101 ± 12	110 ± 11	9.8 ± 0.4	9.2 ± 0.6
	(5)	(5)						
18	38.64 ± 0.69	36.27 ± 0.78*	1555 ± 70	1658 ± 74	96 ± 2	85 ± 12	8.5 ± 0.4	7.8 ± 0.3
	(5)	(6)						

Number of dams used in each group is shown in parentheses.

*P < 0.05 versus pregnant WT mice at the same gestational age, two-tailed Student's *t*-test.

**Table 2:** Primers and probes used for qPCR.

Genes	Primer sequences	GenBank accesion number	Product size (bp)
HPRT	F: 5′-AGCCGACCGGTTCTGTCAT-3′	NM_013556.2	72
	R: 5′-GGTCATAACCTGGTTCATCATCAC-3′		
	Pb: 5′-CGACCCTCAGTCCCAGCGTCGTGAT-3′		
IL-6	F: 5′-CTATACCACTTCACAAGTCGGAGG-3′	NM_031168.1	77
	R: 5′-TGCACAACTCTTTTCTCATTTCC-3		
	Pb: 5′-TTAATTACACATGTTCTCTGGGAAATCG-3′		
NPY	F: 5′-ACAGAAAACGCCCCCAGAAC-3′	NM_023456.2	72
	R: 5′-CGGGAGAACAAGTTTCATTTCC-3′		
	Pb: 5′-AGGCTTGAAGACCCTTCCATGTGG TGAT-3′		
AgRP	F: 5′-ACAACTGCAGACCGAGCAGAA-3′	NM_007427.2	98
	R: 5′-CGACGCGGAGAACGAGACT-3′		
	Pb: 5′-CAGAAGGCAGAAGCTTTGGC GG AGGT-3′		
CART	F: 5′-CGCAT TCCGATCT ACGAGAAGAA-3′	NM_001081493.2	84
	R: 5′-CCT GGCCCCTT TCCTCACT-3′		
	Pb: 5′-CCAAGTCCCCATGTGTGACGCTGGAG-3′.		

HPRT	F: 5′-CAGTCCCAGCGTCGTATT-3	NM_013556.2	139
	R: 5′-AGCAAGTCTTTCAGTCCTGTC-3′		
POMC	F: 5′-TCCATAGACGTGTGAGCTG-3′	NM_139326	174
	R: 5′-GACGTACTTCCGGGGATTTT-3′		
